# Bicomponent Mapping of Cortical Bone Using a New Interleaved UTE Imaging Sequence

**DOI:** 10.1002/mrm.70245

**Published:** 2026-01-04

**Authors:** Soo Hyun Shin, Jiyo S. Athertya, Arya Suprana, James Lo, Jiaji Wang, Dina Moazamian, Fanny Chapelin, Yajun Ma

**Affiliations:** ^1^ Department of Radiology University of California San Diego La Jolla California USA; ^2^ Shu Chien‐Gene Lay Department of Bioengineering University of California San Diego La Jolla California USA

**Keywords:** bicomponent modeling, bone imaging, motion reduction, UTE

## Abstract

**Purpose:**

Ultrashort echo time (UTE) MRI enables direct imaging of cortical bone and quantification of its water compartments via bicomponent T_2_* modeling. However, conventional approaches require multiple separate dual‐echo scans due to limitations in gradient power. This approach is prone to inter‐scan inconsistencies such as motion and signal drift, which degrade fitting accuracy. This study proposes an interleaved dual‐echo acquisition sequence that acquires multiple echo time (TE) images in a single scan to improve bicomponent T_2_* quantification in cortical bone.

**Methods:**

The proposed UTE sequence utilizes interleaved dual‐echo acquisitions with flexible TE spacings. This sequence was tested on five healthy subjects' tibial midshafts and compared to conventional separate dual‐echo scans with and without image registration. Bicomponent T_2_* modeling was performed, and fitting accuracy was evaluated using normalized‐root‐mean‐squared error (NRMSE). Three subjects were scanned three times to evaluate the scan repeatability.

**Results:**

The interleaved method significantly reduced NRMSE (3.2% ± 2.3% vs. 6.2% ± 3.1%, *p* = 0.0231) and yielded lower and more stable T_2_* (T_2s_*; 0.50 ± 0.10 ms vs. 0.76 ± 0.13 ms, *p* < 0.0001) and fraction (F_s_; 78.2 ± 5.1 vs. 84.2% ± 7.1%, *p* = 0.0006) of short T_2_ components compared to separate scans without registration. Image registration had a minimal improvement on mapping results for separate scans. Parameter maps from the interleaved scans confirmed more homogeneous distributions of T_2s_* and F_s_ with lower fitting errors. The much lower coefficients of variance of the interleaved scans demonstrated improved repeatability compared with separate scans.

**Conclusion:**

The proposed interleaved UTE dual‐echo sequence improves the robustness of bicomponent T_2_* mapping of the cortical bone by reducing inter‐scan inconsistencies.

## 1 Introduction

Current bone health assessments primarily rely on X‐ray‐based techniques, such as dual‐energy X‐ray absorptiometry, to measure bone mineral density (BMD). However, minerals make up only about 40% of bone composition, while other critical components—such as collagen fibrils and water—also play essential roles in bone mechanics and fragility [1]. Since BMD does not account for these non‐mineral components, it explains only 30%–50% of osteoporosis cases [2, 3]. This limitation is particularly evident in patients with type 2 diabetes, who often exhibit normal or even elevated BMD despite a significantly increased risk of fractures [4]. These gaps highlight the clinical need for noninvasive methods to assess the non‐mineral phase of bone and provide a more comprehensive evaluation of bone health.

Ultrashort echo time (UTE)‐MRI enables the direct acquisition of short T_2_ bone signals, offering a valuable tool for assessing the organic matrix and water distribution in bone [5, 6]. Beyond morphological imaging of cortical bone, UTE‐MRI allows for the quantitative evaluation of MR relaxation properties, porosity, water composition (e.g., free and bound water pools), and collagen content [7, 8]. These imaging biomarkers have demonstrated strong correlations with porosity measured via high‐resolution micro‐CT and with bone mechanical properties, suggesting their potential to improve diagnostic accuracy in bone health assessment [9, 10].

Among these quantitative UTE techniques, bicomponent T_2_* analysis enables the quantification of pore water (long T_2_* component) and bound water (short T_2_* component) fractions, along with their respective relaxation times [11, 12, 13]. These metrics provide critical insights into water distribution and porosity in cortical bone, both of which influence bone fragility [14, 15]. Bae et al. found that water pool fractions measured by bicomponent modeling are significantly correlated to the mechanical properties of human cortical bones [14]. Similarly, Jerban et al. reported that bicomponent modeling is sensitive to variations in the sizes of the pores in the cortical bone samples [16]. However, due to the inherently short T_2_* of cortical bone, multiple short echo data (< 1 ms) are required for precise bicomponent modeling. Acquiring these short echo data within the same repetition time (TR) is challenging due to the relatively long echo spacings caused by the limited maximum gradient amplitude in clinical scanners. Multiple separate dual‐echo scans with different short echo times (TEs) are typically performed to circumvent this issue. However, this approach is often susceptible to inter‐scan inconsistencies such as subject motion and signal drift, which may compromise bicomponent fitting accuracy.

To overcome this challenge, we developed a novel interleaved UTE sequence that acquires multiple short echo images within a single scan. This sequence incorporates interleaved dual‐echo acquisitions with flexibly chosen short TEs, acquiring multiple TE images in a single scan and potentially avoiding inter‐scan inconsistencies commonly seen in separate dual‐echo acquisitions. We applied the proposed sequence to image the tibial midshafts of healthy volunteers and evaluated its performance in bicomponent T_2_* modeling by comparing the results to those obtained from conventional separate dual‐echo acquisitions, with and without image registration.

## 2 Methods

### 2.1 Sequence Design

**FIGURE 1:**
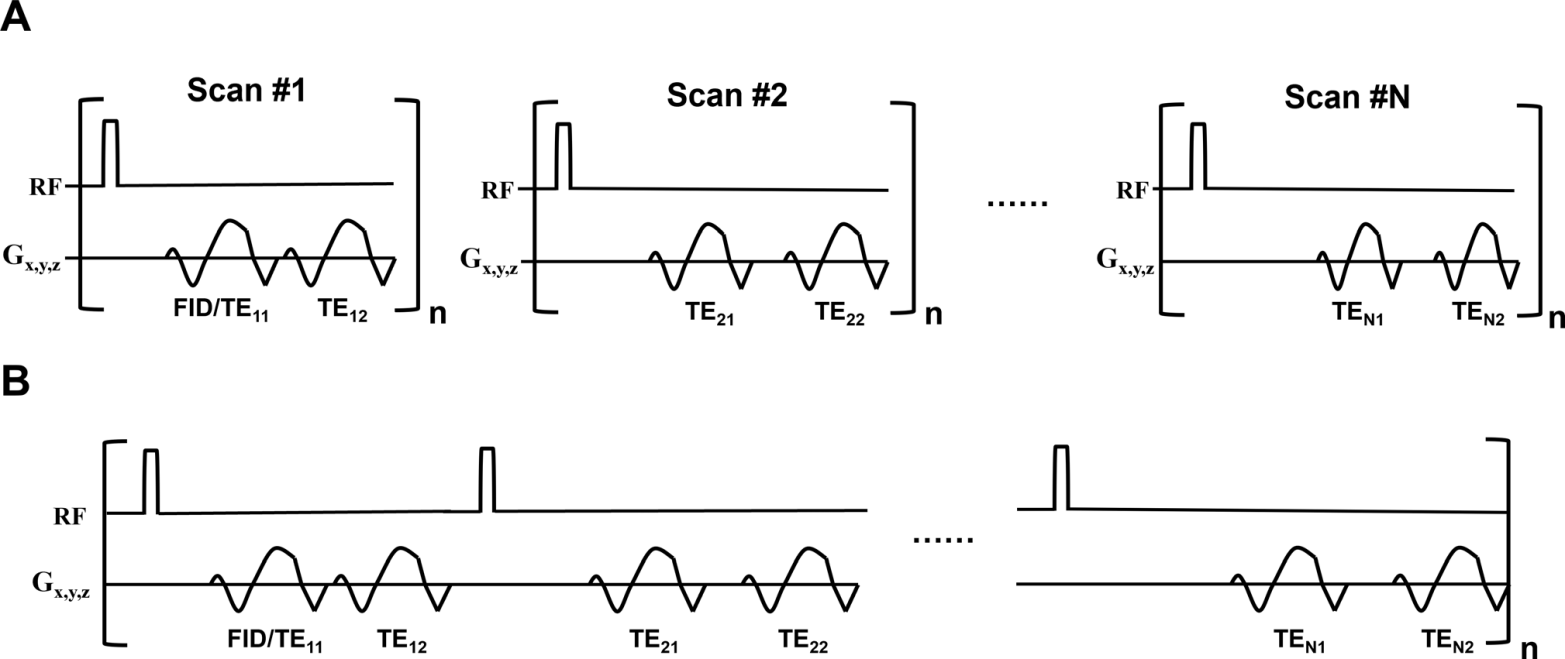
(A) Conventional 3D UTE dual‐echo cones acquisition method, in which multiple separate scans (Scan #1 to #N) are used to obtain images with varying echo times. Each scan acquires a unique dual‐echo pair (TE_ij_, i.e., jth echo of the ith scan), with “*n*” representing the number of spokes needed for fully sampled *k*‐space. (B) Proposed interleaved UTE dual‐echo sequence with flexibly chosen echo times to generate a series of images with different short echo times in a single scan.

In contrast to conventional separate UTE dual‐echo scans for multiple echo acquisitions (Figure 1A), the proposed sequence employs interleaved dual‐echo acquisitions within a single scan (Figure 1B). The TEs can be flexibly chosen by separately controlling the delay time between each RF pulse and readout gradients. A center‐out encoding scheme with a 3D cones trajectory was utilized for time‐efficient k‐space coverage [17, 18].

### 2.2 In Vivo Scan

All the MR experiments were done under the approval of the institutional review board (IRB), with signed consent from volunteers. The new sequence was implemented on a 3 T clinical MRI scanner (MR750, GE Healthcare, Milwaukee, WI, USA), and the tibial midshafts of five healthy subjects (3 male, age = 30.3 ± 6.1 years; 2 female, age = 30.0 ± 8.5 years) were scanned with an 8‐channel knee coil using the following parameters: TR = 12.6 ms, TE = 0.05/2.2, 0.3/4.4, 0.6/6.6, and 0.8/8.8 ms, flip angle (FA) = 10°, FOV = 14 × 14 cm, matrix = 128 × 128, slice thickness = 3 mm, slice number = 32, bandwidth = 250 kHz, and scan time = 7 min 12 s. The short TEs (< 1 ms) in UTE were chosen to capture sufficient information from the rapidly decaying short T_2_ water component for accurate bi‐component fitting, based on the previously reported measurements of T_2_* of the short T_2_ component (0.2–0.6 ms) [5, 11, 14]. For comparison, conventional separate dual‐echo scans (Scan #1: TE = 0.05/2.2 ms, Scan #2: 0.3/4.4 ms, Scan #3: 0.6/6.6 ms, Scan #4: 0.8/8.8 ms) with the same sequence parameters were also performed. The scan time of each dual‐echo acquisition was 1 min 48 s; the total scan time of 4 acquisitions was 7 min 12 s, the same as that of the interleaved acquisition. Three healthy subjects were scanned three times to assess the repeatability of the technique.

### 2.3 Ex Vivo Scan

To investigate whether the discrepancy between the in vivo results from the interleaved and separate scans is due to motion, an ex vivo bovine cortical bone scan was performed. A bovine cortical bone sample was placed in saline and scanned twice (interleaved and separate dual‐echo scans) using the same hardware setup. The scan parameters are as follows: TR = 12.8 ms, FA = 10°, slice thickness = 5 mm, slice number = 24, FOV = 12 × 12 cm, matrix = 128 × 128, and oversampling factor = 2. The selection of TEs is the same as in vivo scans.

### 2.4 Frequency and Signal Drift Assessment

The effects of frequency and signal drift were assessed by scanning a homogeneous phantom using the same hardware setup and sequences as in the in vivo study. The phantom was scanned with (1) four identical separate dual‐echo scans (TE = 0.05/2.2 ms) and (2) an interleaved sequence that acquired four identical echo pairs (TE = 0.05/2.2 ms). Each dual‐echo image was used to calculate a field map by measuring the voxel‐wise phase evolution over the two TEs. The first field map was subtracted from the second and subsequent third and fourth field maps to assess the corresponding frequency drifts. Signal drift was calculated by subtracting the signal intensity of the first image from each subsequent image, then normalizing by the intensity of the first image. The first echo (TE = 0.05 ms) of each scan was used for the signal drift calculation.

### 2.5 Image Registration

The images from separate and interleaved dual‐echo images were co‐registered to account for potential inter‐scan motion. The separately acquired dual‐echo images were registered to the interleaved images through Elastix [19]. Bicomponent T_2_* mapping was performed on unregistered separate dual‐echo scans, registered separate dual‐echo scans, and interleaved scans. The magnitude of translational motion was estimated from the registration process to assess any inter‐scan motion during separate dual‐echo scans.

### 2.6 Data Analysis

**FIGURE 2:**
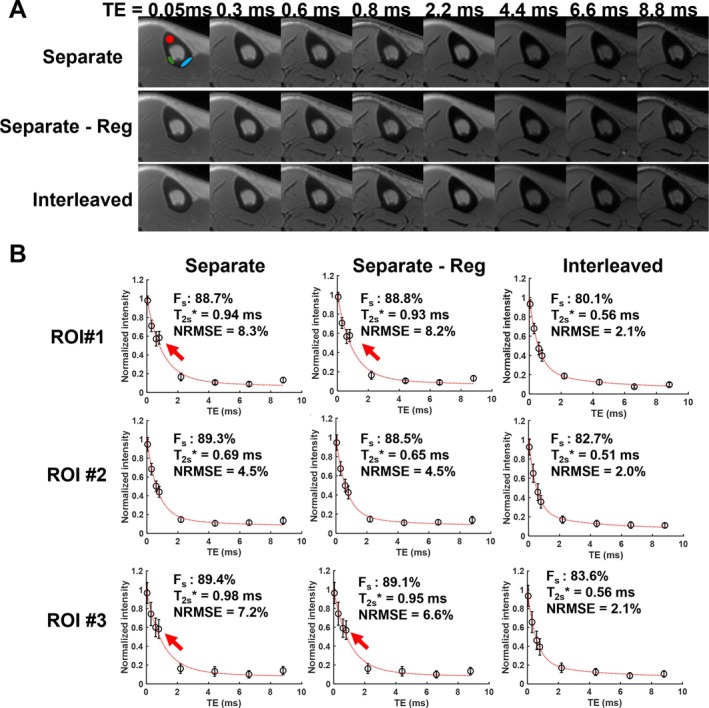
(A) In vivo tibial cortical bone images (29‐year‐old male) obtained using conventional separate dual‐echo acquisitions with (separate‐reg) and without (separate) registration and the proposed interleaved dual‐echo acquisition. Red, blue, and green circles denote regions of interest (ROIs) for analysis, with ROI#1 in the red, ROI#2 in the blue, and ROI#3 in the green circle. (B) Bicomponent T_2_* fitting results for ROIs indicated in (A). The fitting obtained from the interleaved acquisition shows a lower normalized‐root‐mean‐squared error (NRMSE) compared to separate dual‐echo acquisitions, indicating improved accuracy of T_2_* measurements. Red arrows indicate signal oscillations in the T_2_* decay curves from separate dual‐echo scans (T_2s_*: T_2_* of short T_2_ component, F_s_: fraction of short T_2_ component).

Bicomponent fitting was performed using a custom‐made MATLAB code (MATLAB 2024a, MathWorks, Natick, MA, USA). Regions of interest (ROIs) were drawn on the anterior border and the posterior side of the tibial cortical bone (Figure 2A) for bicomponent T_2_* analysis. For the bicomponent T_2_* modeling, the ROI‐averaged signal intensities were fitted to the following biexponential equation:

$$S=S_{0}\left[F_{s}e^{-\mathrm{TE}/T_{2s}*}+\left(1-F_{s}\right)e^{-\mathrm{TE}/T_{2L}*}\right]+\text{noise}$$

*S* and *S*_0_ denote the measured signal intensities and the signal at TE = 0, respectively. T_2s_* and T_2L_* are T_2_*s of short and long T_2_ water components, respectively. F_s_ is the fraction of the short T_2_ component. The noise term was measured as a mean signal intensity in a 20 × 20‐voxel ROI placed in the background (air) of the last echo image. The trust‐region algorithm was used for the non‐linear fitting.

To potentially increase the robustness of the fitting, the T_2L_* was fixed to 4 ms based on previous studies [11]. Fitting without fixing T_2L_* was also performed for comparison. Fitting accuracy was evaluated using normalized‐root‐mean‐squared error (NRMSE). For ex vivo bovine bone sample, the fixed T_2L_* value was varied from 3 to 6 ms during the fitting to assess the robustness of the bicomponent modeling against the varying T_2L_* values.

### 2.7 Statistical Analysis

The coefficient of variance (CoV) was used as a metric of repeatability measurement for F_s_ and T_2s_*, as well as a measurement of the robustness of voxel‐wise fitting. The average of six ROI‐based measurements over three slices (2 ROIs per slice) was used as a representative measurement of each repeat scan. For further assessment of repeatability, Bland–Altman plots were also generated with measurements from eight additional ROIs per subject per scan. The T_2s_* and F_s_ measurements, as well as fitting errors and standard deviations of voxel‐wise measurements from interleaved and separate dual‐echo images with and without registration, were compared using one‐way ANOVA with Tukey's post hoc test. The normal distribution of the data was confirmed via the Shapiro–Wilk test. All the data points are represented as mean ± SD. All the statistical analyses were done using Prism 10.2 (GraphPad, Boston, MA, USA).

## 3 Results

The images acquired from the proposed interleaved dual‐echo scans and conventional separate dual‐echo scans, regardless of whether registered or not, show minimal differences visually (Figure 2A). However, notable differences appear in the ROI‐based bicomponent T_2_* fitting results. Pronounced signal oscillations are observed in the T_2_* decay curves from separate dual‐echo scans compared to those from interleaved scans (Figure 2B), resulting in higher fitting errors from the separate dual‐echo scans. These oscillations also lead to overestimated T_2s_* and F_s_ in separate dual‐echo scans. A similar trend of signal oscillations and ROI‐based measurements from another subject is also shown in Figure S1.

**FIGURE 3:**
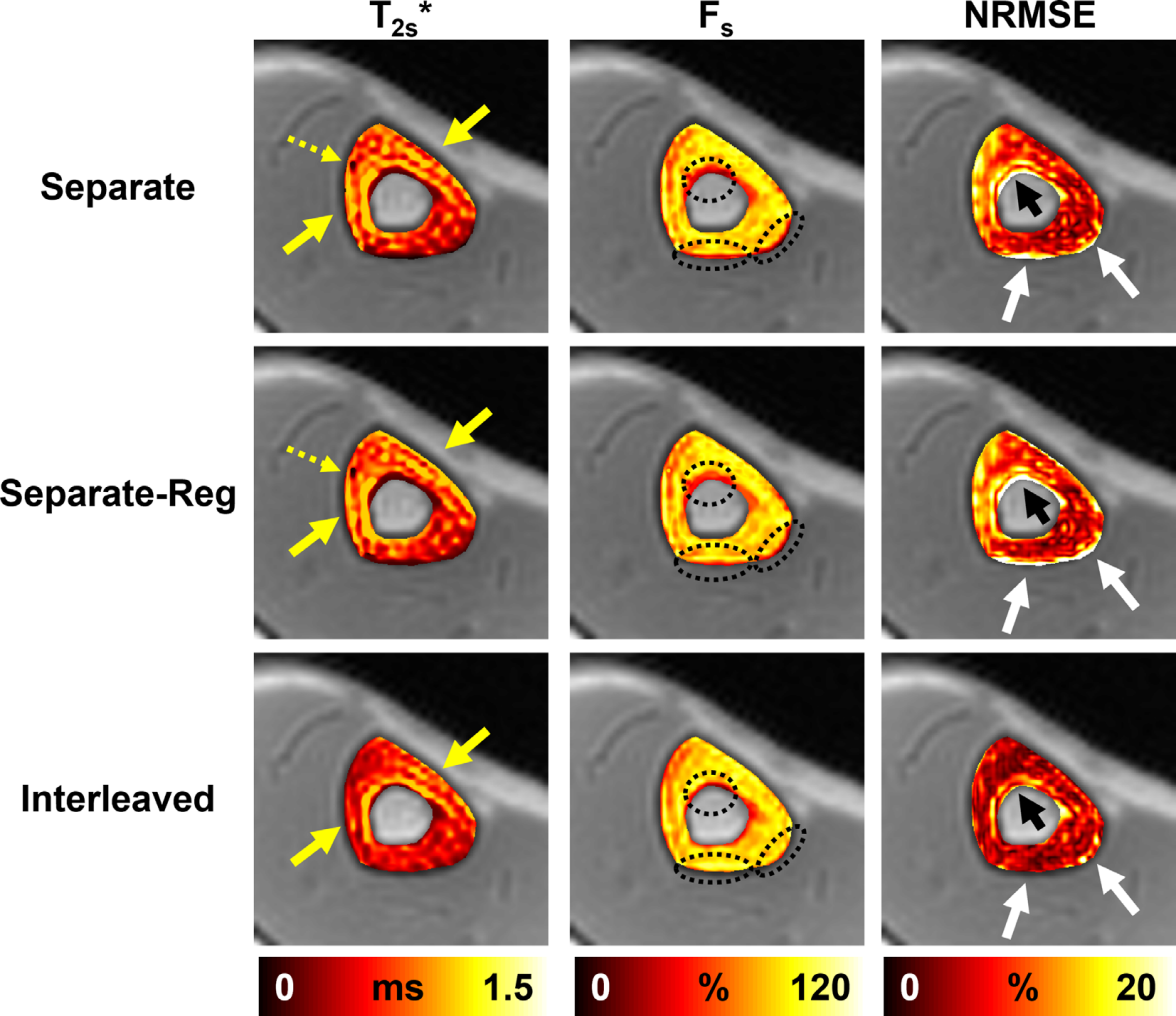
Bicomponent parameter maps of the tibial midshaft cortical bone of a 37‐year‐old male obtained from conventional separate dual‐echo scans with (separate‐reg) and without (separate) registration and interleaved dual‐echo scan. The fitting error map (normalized‐root‐mean‐squared, NRMSE) demonstrates lower fitting errors with the proposed interleaved scan, with marked errors from separate scans in the periosteum (white arrows). These fitting errors are reflected in the T_2s_*, in which yellow arrows indicate overestimated T_2s_* regions in the separate dual‐echo acquisitions compared to the interleaved scan. Yellow dotted arrows indicate unstable T_2s_* measurements from separate scans. The difference in F_s_ measurements from separate and interleaved scans is also shown in the marrow–bone interface (black dotted circle).

**FIGURE 4:**
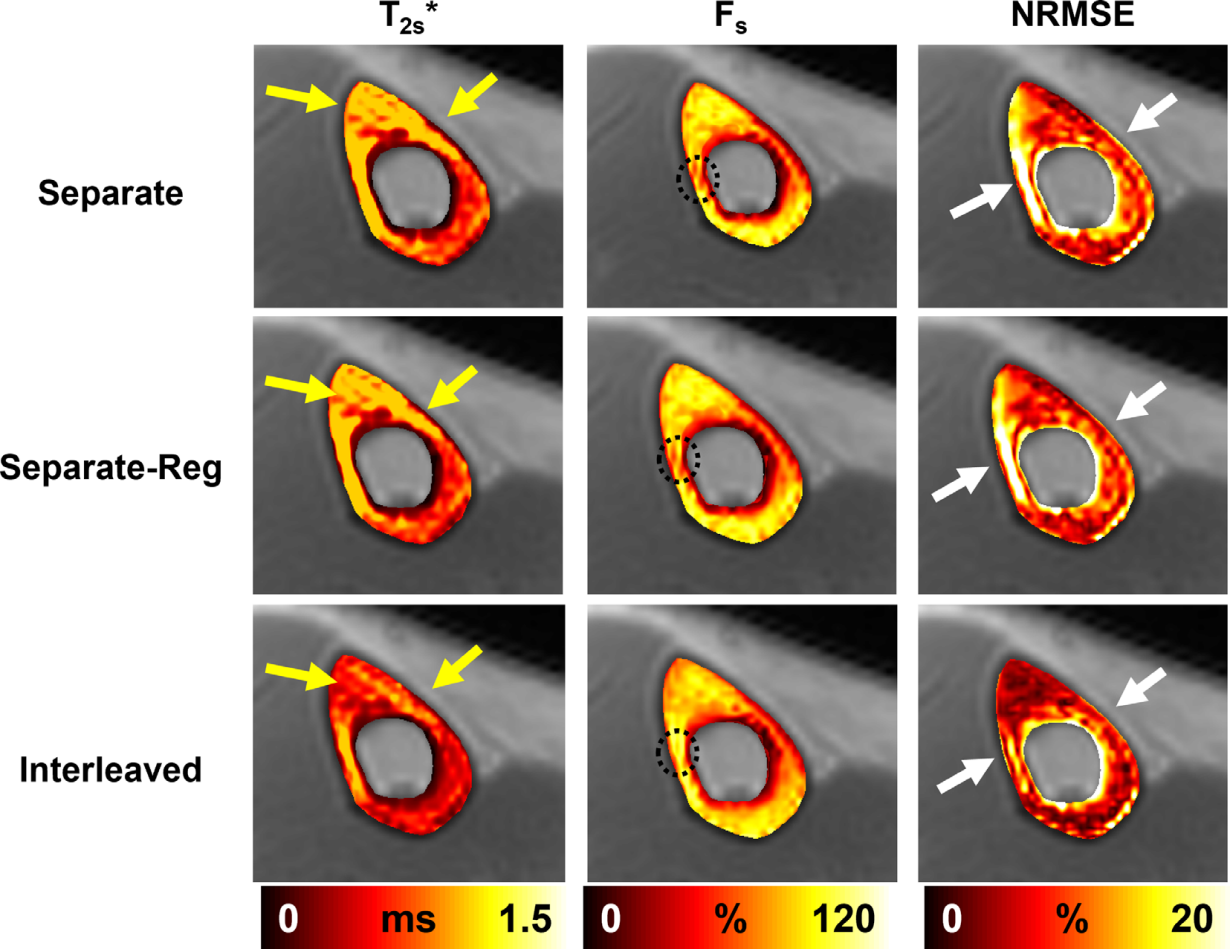
Bicomponent parameter maps of the tibial cortical bone of a 29‐year‐old male obtained from conventional separate dual‐echo scans with (separate–reg) and without (separate) registration and a single interleaved dual‐echo scan. The fitting error map (normalized‐root‐mean‐squared, NRMSE) demonstrates lower fitting errors with the proposed interleaved scan. Other parameter maps also exhibit more homogeneous and stable measurements in the interleaved scan compared to separate scans. Yellow arrows indicate the medial and anterior sides of the bone where T_2s_* measurements are markedly different between separate and interleaved scans. The difference of F_s_ measurements from separate and interleaved scans is more evident in tissue boundaries (black dotted circles), which is also reflected in the NRMSE maps (white arrows).

These findings are consistent with voxel‐wise parameter mapping, which shows relatively shorter T_2s_* values and lower NRMSEs in the interleaved scans. Representative parameter maps from two subjects are presented in Figures 3 and 4. The F_s_ maps from the interleaved and separate scan methods are largely similar; however, discrepancies are observed, primarily at bone‐muscle and bone‐fat boundaries (dotted circles in Figures 3 and 4). These differences are also evident in the NRMSE maps, which show higher fitting errors at these tissue interfaces in the separate dual‐echo scans (white arrows in Figures 3 and 4). These pronounced boundary differences suggest that measurements from separate dual‐echo scans are affected by subtle inter‐scan motion, which could not be fully corrected by post‐image registration. In T_2s_* mapping, the separate dual‐echo scans—with and without image registration—exhibit greater regional variations, including elevated T_2s_* values along the anterior border and medial‐lateral sides (yellow arrows), compared to the interleaved scan. Additionally, the separate scan method shows voxels with unstable fitting, resulting in abnormally low T_2s_* values (yellow dotted arrows). These voxels with unstable fitting results may arise from artifacts or signal fluctuations that cause non‐exponential behavior of the signal decay, such as partial volume effect, hardware instability, and motion. In contrast, the T_2s_* maps from the interleaved scan demonstrate a more homogeneous distribution throughout the cortical bone. Statistical comparison of spatial variations of bicomponent model parameters also shows significantly reduced variations in T_2s_* (*p* = 0.0342) measured from the interleaved scan compared to those from separate dual‐echo scans (Figure S2).

Figure S3 presents the mapping results without fixing T_2L_* in the fitting process. Compared to results from the same subject with a fixed T_2L_* (Figure 3), there is a substantial increase in the number of voxels reaching the lower and upper limits of the nonlinear fitting across all three parameter maps (T_2s_*, F_s_, and T_2L_*). The unstable fitting is also evident in the CoVs of the voxel‐wise measurements (Table S1) that show increased CoVs from the measurements without fixing T_2L_*. Nevertheless, the patterns of discrepancy between the interleaved and separate scans—such as overestimated T_2s_* values and higher fitting errors at tissue boundaries in the separate scans—remain consistent with those observed in Figure 3.

**FIGURE 5:**
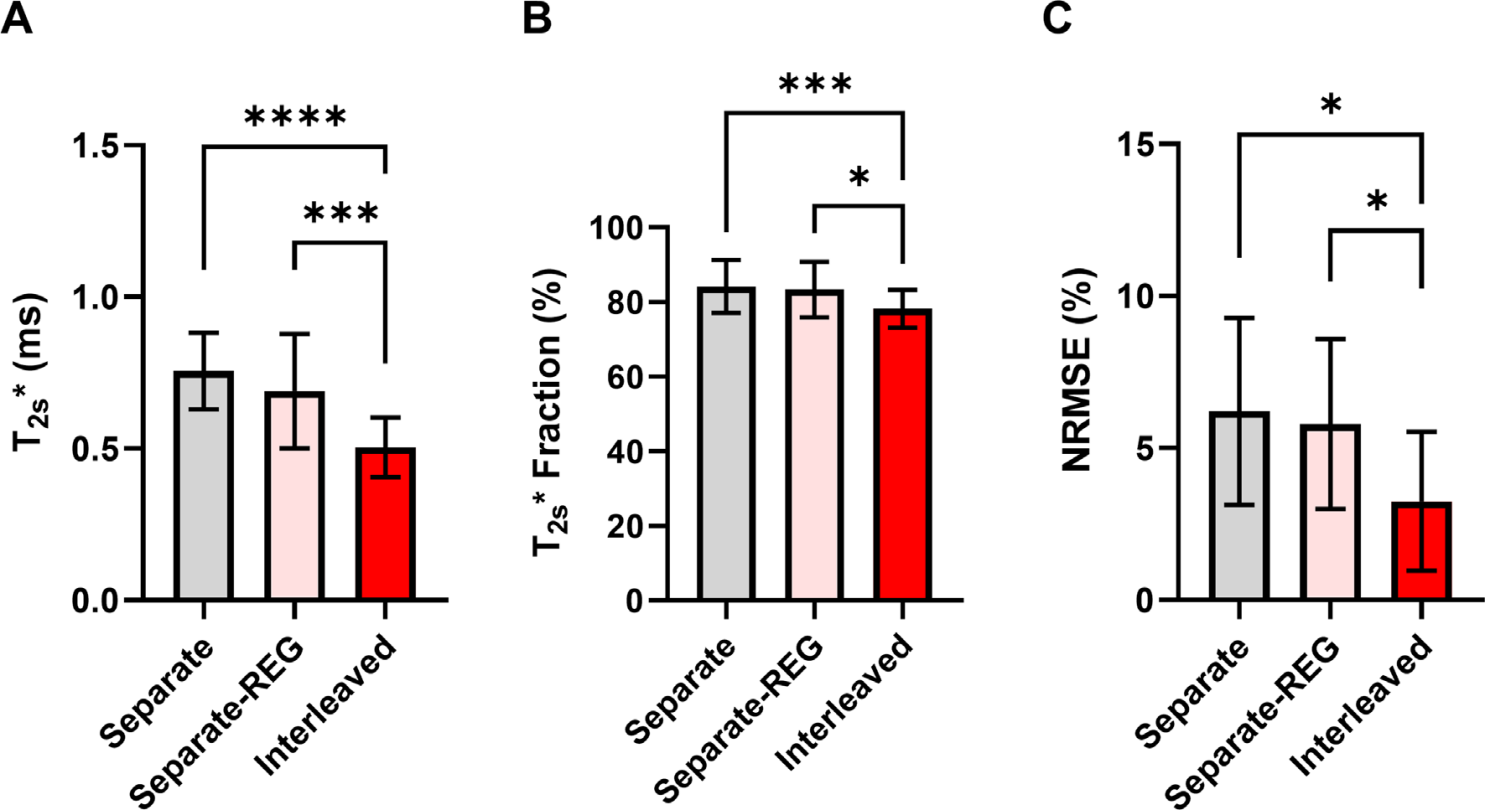
Summary of ROI‐based measurements from five healthy volunteers: (A) T_2_* of short T_2_ component (T_2s_*), (B) fraction of short T_2_ component (F_s_), and (C) fitting error measured by normalized‐root‐mean‐squared error (NRMSE). Measurements from the proposed single interleaved scan exhibit significantly (A) lower T_2s_* (B) lower F_s_, and (C) lower NRMSE, compared to separate dual‐echo scans (*p < 0.05, ***p < 0.001, ****p < 0.0001).

Figure 5 summarizes the bicomponent measurements from five volunteer scans. The intersubject standard deviations of T_2s_* and F_s_ are notably lower in interleaved scans (T_2s_*: 0.10 ms, F_s_: 5.1%) compared to separate dual‐echo scans with (T_2s_*: 0.19 ms, F_s_: 7.4%) and without registration (T_2s_*: 0.13 ms, F_s_: 7.1%). Consistent with findings in Figures 2, 3, 4, the interleaved scans yield significantly shorter T_2s_* values (0.50 ± 0.10 ms) than the separate scans without registration (0.7 ± 0.13 ms, *p* < 0.0001) and with registration (0.69 ± 0.19 ms, *p* = 0.001; Figure 5A). Similarly, F_s_ is significantly lower in the interleaved scans (78.2% ± 5.1%) compared to the separate scans without registration (84.2% ± 7.1%, *p* = 0.0006; Figure 5B) and to the registered separate scans (83.4% ± 7.4%, *p* = 0.0202). The interleaved scans also show significantly lower fitting errors, as indicated by NRMSE (3.2% ± 2.3%), compared to the separate scans with (5.8% ± 2.8%, *p* = 0.0321) and without registration (6.2% ± 3.1%, *p* = 0.0231; Figure 5C).

Ex vivo bovine bone scan shows significantly reduced difference between bicomponent modeling results generated from interleaved and separate dual‐echo scans (Figure S4). The mean voxel‐wise relative differences of T_2s_* and F_s_ between two scans were 5.3% and 1.6%, respectively. Compared to the ROI‐based measurements from in vivo scans, which showed approximately 28.6% difference in T_2s_* (0.5 vs. 0.7 ms) and 7.1% difference in F_s_ (78.2% vs. 84.2%) between interleaved and unregistered separate dual‐echo scans, the relative differences shown in the bovine bone sample are substantially lower. The ex vivo bovine bone data were also used to assess the sensitivity of the bicomponent modeling procedure with varying the fixed values of T_2L_*. With the fixed T_2L_* varying from 3 to 6 ms, the T_2s_* and F_s_ measurements showed variations of less than 10% compared to the results from T_2s_* fixed to 4 ms (Figure S5).

The phantom study shows that separate dual‐echo scans are subject to frequency and signal drift, whereas the interleaved sequence is less sensitive to these effects (Figure S6). Over the four separate dual‐echo scans, ∼2 Hz frequency drift is observed (total 7 min 12 s), which is consistent with the previously reported 6 Hz drift over 24 min of UTE scans [20]. Along with this frequency drift, ∼1.2% signal drift is also observed. The interleaved scans also show minimal frequency and signal drift between the first and subsequent echo pairs.

The repeatability study further supports the robustness of the proposed interleaved scan strategy, showing lower mean CoVs across the three subjects in interleaved scans for both T_2s_* (4.1% ± 2.1%) and F_s_ (3.6% ± 2.0%), compared to separate scans with (T_2s_*: 9.0% ± 3.4%, F_s_: 4.5% ± 3.0%) and without registration (T_2s_*: 11.5% ± 1.4%, F_s_: 4.5% ± 3.0%). The Bland–Altman plots drawn with multiple ROI‐based measurements show the consistency of bicomponent parameter measurements with reduced inter‐scan differences shown in interleaved scans (Figure S7).

## 4 Discussion

The proposed interleaved dual‐echo UTE sequence provided more reliable bicomponent T_2_* measurements than conventional separate dual‐echo scans. It significantly reduced biexponential fitting errors and minimized regional variations in voxel‐wise T_2s_* values and F_s_ measurements. The interleaved approach also showed higher repeatability compared to separate dual‐echo scans. These improvements demonstrate that interleaved UTE acquisition is a promising technique for probing different water pools in cortical bone in vivo with enhanced accuracy and robustness.

Conventional separate dual‐echo scans are prone to inter‐scan motion. Although separate dual‐echo scans also use the center‐out trajectory that reduces motion sensitivity, it is insensitive only to the motion that occurs within each individual scan. Any motion that occurs between independent scans cannot be suppressed and requires a retrospective registration process. This inter‐scan motion is particularly problematic for cortical bone, considering that low spatial resolution is often used to overcome its inherently low SNR. The low‐resolution results in a small number of voxels in the cortical bone, so that any small motions may lead to fitting errors. We observed 0.3–0.7 mm translational motion in separate dual‐echo scans via Elastix registration. These sub‐voxel motions may also affect quantitative analysis due to the partial volume effect [21, 22]. It should also be noted that these image registration algorithms do not always fully compensate for motion artifacts, potentially introducing errors in the analysis [23]. Our results indicate that post‐image registration did not significantly improve bicomponent T_2_* fitting, suggesting the residual motion artifacts. In comparison, the proposed interleaved dual‐echo acquisition converts all the inter‐scan motions into intra‐scan motions, thereby benefiting from the reduced motion sensitivity of the center‐out trajectory. Center‐out trajectories have been extensively used for free‐breathing abdominal imaging and subsequent quantitative measurements of R_2_*, fat, and iron deposition in the liver [24, 25, 26]. These studies demonstrated that intra‐scan motion artifacts are substantially mitigated by the center‐out acquisition strategy. Given that motion in the tibial midshaft is considerably smaller than in abdominal tissues, it is highly likely that redistributing all motion from inter‐scan to intra‐scan is advantageous compared with performing repeated shorter scans—even with the longer effective scan time in interleaved acquisitions. The reduced discrepancy between the results from interleaved and separate dual‐echo scans of an inanimate ex vivo bovine bone sample also supports that the inter‐scan motion is a primary source that causes differences in bicomponent modeling results.

Another potential source of inter‐scan inconsistency that may interfere with the bicomponent analysis is center frequency and signal drift due to MR hardware heating, as shown in previous studies on 3D radial UTE, diffusion MRI, and EPI experiments [27, 28, 29]. Similar to these previous studies, we observed a 2 Hz frequency shift and 1.2% signal drift from separate dual‐echo scans. Considering this small frequency shift is almost negligible, we don't expect that this drift would affect our bicomponent modeling significantly.

To improve the robustness of the bicomponent modeling, we fixed T_2L_* to 4 ms [11] during the fitting process to reduce the degree of freedom and corresponding variability. The increased robustness is evident by comparing Figure 3 and Figure S1, which show the voxel‐wise fitting results from the same subject with and without fixing the T_2L_*, respectively. The smaller CoV of the voxel fitting results after fixing T_2L_* compared to those without fixing T_2L_* also demonstrates the increased robustness of the fitting. Ex vivo bovine bone scan also showed that a variation of the fixed value of T_2L_* within the reasonable range leads to minimal variations in T_2s_* and F_s_ measurements, further supporting the usage of fixed T_2L_* for bicomponent modeling. Combined with the proposed interleaved UTE sequence, this strategy of fixing T_2L_* may enable more reliable fitting, facilitating clinical translation of the bicomponent modeling approach.

The robustness of the fitting also depends on the SNR of the images. Our simulation of ROI‐based fitting (data not shown) indicated that processing data with an SNR below 30 results in significant variation and bias in the fitting results. Since the approximate SNR of the cortical bone scanned in this study is 50, we expect negligible bias in our measurements.

While our interleaved approach was tested specifically on tibial midshafts using bicomponent T_2_* modeling, this method can be extended to other tissues containing short T_2_ components and other quantitative MRI techniques requiring multiple echo acquisitions. Bicomponent T_2_* modeling has been used to investigate tissues such as tendons, menisci, cartilage, and ligaments to assess different water compartments [12, 30, 31, 32]. For example, this technique has demonstrated the potential for diagnosing tendinopathy by identifying decreased F_s_ in the patella [33]. Another study reported a significant negative correlation between Achilles tendon rupture scores and T_2s_* values [34]. Additionally, bicomponent T_2_* modeling can be expanded into a tricomponent model to account for residual fat in cortical bone [35, 36]. Since increased bone marrow adiposity and trabecularization of cortical bone are commonly associated with skeletal and metabolic disorders—including osteoporosis [37], diabetes [38], and chronic kidney disease [39]—monitoring both water compartments and fat content in cortical bone could enhance our understanding of bone pathophysiology and improve diagnostic accuracy [40]. Given that any artifacts become increasingly critical when using more complex signal models, our interleaved sequence will be valuable for the clinical translation of multicomponent T_2_* modeling approaches. Yet, this multicomponent modeling comes with an increased degree of freedom, requiring data with more TEs to be acquired with the chemical shift between different pools taken into account.

Several limitations of our technique still need to be addressed. As a proof‐of‐concept study, we tested our sequence on only five healthy subjects. A larger cohort is needed to validate the robustness of this technique. Moreover, this interleaved technique should be evaluated in patients with bone disorders to demonstrate its clinical utility in assessing different bone water compartments. Furthermore, future studies should explore the applicability of our technique to other short T_2_ tissues and various bone types throughout the body.

## 5 Conclusion

The proposed interleaved UTE acquisition scheme enables more robust bicomponent T_2_* measurements of the cortical bone of the tibial midshaft compared to conventional separate dual‐echo scans for in vivo study.

## Funding

This work was supported by the National Institutes of Health (R01AR079484).

## Supporting information


**Table S1.** Coefficient of variables (CoV) of the voxel‐wise measurements of T_2_* of the short T_2_ component (T_2s_*) and the fraction of the short T_2_ component (F_s_) with and without fixing the T_2_* of the long T_2_ component (T_2L_*). The voxel‐wise measurements are shown in Figure 3 (fix) and Figure S1 (no fix).
**Figure S1.** ROI‐based Bicomponent T_2_* fitting results from the tibial midshaft cortical bone of a 37‐year‐old male shown in Figure 3. Red arrows indicate signal oscillations from separate dual‐echo scans with and without registration.
**Figure S2.** Comparison of standard deviations of voxel‐wise measurements of (A) T_2s_* fraction and (B) T_2s_* from interleaved and separate dual‐echo scans. Interleaved scan shows reduced variances in voxel‐wise mapping of bicomponent model parameters in general. The reduction of variances in the T_2s_* fraction measurement from the interleaved scan is statistically significant (*p* = 0.0342) compared to the results from separate scans.
**Figure S3.** Bicomponent parameter maps from a 37‐year‐old male healthy subject's tibial midshaft with unfixed long T_2_* (T_2L_*) for fitting. In the bicomponent fitting, the lower and upper boundary of T_2L_* was set to 0 and 10 ms, respectively. Compared to the fitting results with fixed T_2L_* of 4 ms shown in Figure 3, the overall range of fitting results is similar but more unstable fitting. Nevertheless, the interleaved sequence still shows more stable fitting results compared to the separate dual‐echo acquisition with (Separate‐Reg) and without (Separate) image registration (T_2s_* = T_2_* of short T_2_ component, F_s_ = Fraction of short T_2_ component).
**Figure S4.** Voxel‐wise bicomponent fitting results from a bovine cortical bone scan. The separate dual‐echo and interleaved scans yield highly consistent and relatively homogeneous T_2s_* and F_s_ (T_2s_* fraction) measurements. The relative difference maps, along with the mean relative differences between the two scans, are also shown. Notably, the T_2s_* (5.3%) and F_s_ (1.6%) differences are substantially lower than the relative differences observed in vivo (T_2s_*: ∼28%; F_s_: ∼7%).
**Figure S5.** Voxel‐wise bicomponent fitting of the interleaved ex vivo bovine bone scan with varying values of fixed T_2L_*. The differences were computed by subtracting the fitting results obtained with T_2L_* = 4 ms from those using other tested values, and then normalizing by the T_2L_* = 4 ms result. Overall, F_s_ and T_2s_* vary by less than ±10% when T_2L_* is varied from 3 to 6 ms, which is consistent with typical observations reported in previous bicomponent analysis studies.
**Figure S6.** Evaluation of frequency and signal drift in interleaved and separate dual‐echo scans. (A) The maps of frequency (left) and signal difference (right) between the first acquisition (Rep 1) and subsequent repeated scans (Rep 2, 3, 4) of a homogenous phantom. Note that the interleaved acquisition shows a consistent frequency and signal intensity between the first image and the subsequent repeat scans, whereas increasing frequency and signal differences over repeats are evident in separate dual‐echo scans. (B) The signal drift measurements from an ROI encompassing the entire phantom.
**Figure S7.** Bland–Altman plots of (A) T_2s_* and (B) F_s_* fraction measurements from repeat scans of three subjects. Dashed lines indicate ±1.96 standard deviation. The fitted results from interleaved and separate dual‐echo scans with and without registration show the measurements among repeat scans align well. The interleaved scans show smaller variations in the differences between the repeat scans compared to the separate dual‐echo scans.

## References

[1] E. F. Morgan , G. U. Unnikrisnan , and A. I. Hussein , “Bone Mechanical Properties in Healthy and Diseased States,” Annual Review of Biomedical Engineering 20, no. 1 (2018): 119–143, 10.1146/annurev-bioeng-062117-121139. PMC6053074 29865872

[2] K. G. Faulkner , “Bone Matters: Are Density Increases Necessary to Reduce Fracture Risk?,” Journal of Bone and Mineral Research 15, no. 2 (2000): 183–187, 10.1359/jbmr.2000.15.2.183. 10703919

[3] S. C. E. Schuit , M. van der Klift , A. E. A. M. Weel , et al., “Fracture Incidence and Association With Bone Mineral Density in Elderly Men and Women: The Rotterdam Study,” Bone 34, no. 1 (2004): 195–202, 10.1016/j.bone.2003.10.001. 14751578

[4] A. V. Schwartz , E. Vittinghoff , D. C. Bauer , et al., “Association of BMD and FRAX Score With Risk of Fracture in Older Adults With Type 2 Diabetes,” Journal of the American Medical Association 305, no. 21 (2011): 2184–2192, 10.1001/jama.2011.715. 21632482 PMC3287389

[5] J. Du , M. Carl , M. Bydder , A. Takahashi , C. B. Chung , and G. M. Bydder , “Qualitative and Quantitative Ultrashort Echo Time (UTE) Imaging of Cortical Bone,” Journal of Magnetic Resonance 207, no. 2 (2010): 304–311, 10.1016/j.jmr.2010.09.013. 20980179

[6] S. H. Shin , H. D. Chae , A. Suprana , et al., “UTE MRI Technical Developments and Applications in Osteoporosis: A Review,” Frontiers in Endocrinology 16 (2025): 1510010, 10.3389/fendo.2025.1510010. 39980853 PMC11839439

[7] Y. J. Ma , S. Jerban , H. Jang , D. Chang , E. Y. Chang , and J. Du , “Quantitative Ultrashort Echo Time (UTE) Magnetic Resonance Imaging of Bone: An Update,” Frontiers in Endocrinology 11 (2020): 11, 10.3389/fendo.2020.567417. 33071975 PMC7531487

[8] S. Jerban , Y. Ma , Z. Wei , et al., “Ultrashort Echo Time MRI Detects Significantly Lower Collagen but Higher Pore Water in the Tibial Cortex of Female Patients With Osteopenia and Osteoporosis,” Journal of Bone and Mineral Research 39, no. 6 (2024): 707–716, 10.1093/jbmr/zjae053. 38591788 PMC11523241

[9] S. Jerban , Y. Ma , L. Li , et al., “Volumetric Mapping of Bound and Pore Water as Well as Collagen Protons in Cortical Bone Using 3D Ultrashort Echo Time Cones MR Imaging Techniques,” Bone 127 (2019): 120–128, 10.1016/j.bone.2019.05.038. 31176044 PMC6708764

[10] S. Jerban , Y. Ma , E. W. Dorthe , et al., “Assessing Cortical Bone Mechanical Properties Using Collagen Proton Fraction From Ultrashort Echo Time Magnetization Transfer (UTE‐MT) MRI Modeling,” Bone Reports 11 (2019): 100220, 10.1016/j.bonr.2019.100220. 31440531 PMC6700521

[11] L. Li , Y. Chen , Z. Wei , et al., “3D UTE Bicomponent Imaging of Cortical Bone Using a Soft–Hard Composite Pulse for Excitation,” Magnetic Resonance in Medicine 85, no. 3 (2021): 1581–1589, 10.1002/mrm.28528. 32989787 PMC7924727

[12] J. Du , E. Diaz , M. Carl , W. Bae , C. B. Chung , and G. M. Bydder , “Ultrashort Echo Time Imaging With Bicomponent Analysis,” Magnetic Resonance in Medicine 67, no. 3 (2012): 645–649, 10.1002/mrm.23047. 22034242

[13] S. Li , E. Y. Chang , W. C. Bae , et al., “The Effect of Excitation and Preparation Pulses on Nonslice Selective 2D UTE Bicomponent Analysis of Bound and Free Water in Cortical Bone at 3T,” Medical Physics 41, no. 2 (2014): 022306, 10.1118/1.4862838. 24506644 PMC3987690

[14] W. C. Bae , P. C. Chen , C. B. Chung , K. Masuda , D. D'Lima , and J. Du , “Quantitative Ultrashort Echo Time (UTE) MRI of Human Cortical Bone: Correlation With Porosity and Biomechanical Properties,” Journal of Bone and Mineral Research 27, no. 4 (2012): 848–857, 10.1002/jbmr.1535. 22190232 PMC3463505

[15] J. Du , J. C. Hermida , E. Diaz , et al., “Assessment of Cortical Bone With Clinical and Ultrashort Echo Time Sequences: Imaging of Cortical Bone,” Magnetic Resonance in Medicine 70, no. 3 (2013): 697–704, 10.1002/mrm.24497. 23001864

[16] S. Jerban , Y. Ma , J. H. Wong , et al., “Ultrashort Echo Time Magnetic Resonance Imaging (UTE‐MRI) of Cortical Bone Correlates Well With Histomorphometric Assessment of Bone Microstructure,” Bone 123 (2019): 8–17, 10.1016/j.bone.2019.03.013. 30877070 PMC6504977

[17] P. Irarrazabal and D. G. Nishimura , “Fast Three Dimensional Magnetic Resonance Imaging,” Magnetic Resonance in Medicine 33, no. 5 (1995): 656–662, 10.1002/mrm.1910330510. 7596269

[18] P. T. Gurney , B. A. Hargreaves , and D. G. Nishimura , “Design and Analysis of a Practical 3D Cones Trajectory,” Magnetic Resonance in Medicine 55, no. 3 (2006): 575–582, 10.1002/mrm.20796. 16450366

[19] S. Klein , M. Staring , K. Murphy , M. A. Viergever , and J. P. W. Pluim , “Elastix: A Toolbox for Intensity‐Based Medical Image Registration,” IEEE Transactions on Medical Imaging 29, no. 1 (2010): 196–205, 10.1109/TMI.2009.2035616. 19923044

[20] A. Fischer , P. Martirosian , T. Benkert , and F. Schick , “Spatially Resolved Free‐Induction Decay Spectroscopy Using a 3D Ultra‐Short Echo Time Multi‐Echo Imaging Sequence With Systematic Echo Shifting and Compensation of B0 Field Drifts,” Magnetic Resonance in Medicine 87, no. 5 (2022): 2099–2110, 10.1002/mrm.29115. 34866240

[21] S. Kecskemeti , A. Samsonov , J. Velikina , et al., “Robust Motion Correction Strategy for Structural MRI in Unsedated Children Demonstrated With Three‐Dimensional Radial MPnRAGE,” Radiology 289, no. 2 (2018): 509–516, 10.1148/radiol.2018180180. 30063192 PMC6192848

[22] I. Terem , W. W. Ni , M. Goubran , et al., “Revealing Sub‐Voxel Motions of Brain Tissue Using Phase‐Based Amplified MRI (aMRI),” Magnetic Resonance in Medicine 80, no. 6 (2018): 2549–2559, 10.1002/mrm.27236. 29845645 PMC6269230

[23] M. Zaitsev , J. Maclaren , and M. Herbst , “Motion Artifacts in MRI: A Complex Problem With Many Partial Solutions,” Magnetic Resonance Imaging 42, no. 4 (2015): 887–901, 10.1002/jmri.24850. PMC4517972 25630632

[24] Y. Kee , C. M. Sandino , A. B. Syed , et al., “Free‐Breathing Mapping of Hepatic Iron Overload in Children Using 3D Multi‐Echo UTE Cones MRI,” Magnetic Resonance in Medicine 85, no. 5 (2021): 2608–2621, 10.1002/mrm.28610. 33432613 PMC8886621

[25] T. Armstrong , X. Zhong , S. F. Shih , et al., “Free‐Breathing 3D Stack‐Of‐Radial MRI Quantification of Liver Fat and R2* in Adults With Fatty Liver Disease,” Magnetic Resonance Imaging 85 (2022): 141–152, 10.1016/j.mri.2021.10.016. 34662702

[26] X. Zhong , M. D. Nickel , S. A. R. Kannengiesser , et al., “Accelerated Free‐Breathing Liver Fat and R2* Quantification Using Multi‐Echo Stack‐Of‐Radial MRI With Motion‐Resolved Multidimensional Regularized Reconstruction: Initial Retrospective Evaluation,” Magnetic Resonance in Medicine 92, no. 3 (2024): 1149–1161, 10.1002/mrm.30117. 38650444

[27] W. Jiang , F. Ong , K. M. Johnson , et al., “Motion Robust High Resolution 3D Free‐Breathing Pulmonary MRI Using Dynamic 3D Image Self‐Navigator,” Magnetic Resonance in Medicine 79, no. 6 (2018): 2954–2967, 10.1002/mrm.26958. 29023975 PMC6474413

[28] S. B. Vos , C. M. W. Tax , P. R. Luijten , S. Ourselin , A. Leemans , and M. Froeling , “The Importance of Correcting for Signal Drift in Diffusion MRI,” Magnetic Resonance in Medicine 77, no. 1 (2017): 285–299, 10.1002/mrm.26124. 26822700

[29] T. Benner , A. J. W. van der Kouwe , J. E. Kirsch , and A. G. Sorensen , “Real‐Time RF Pulse Adjustment for B0 Drift Correction,” Magnetic Resonance in Medicine 56, no. 1 (2006): 204–209, 10.1002/mrm.20936. 16767763

[30] H. Jang , A. B. McMillan , Y. Ma , et al., “Rapid Single Scan Ramped Hybrid‐Encoding for Bicomponent T2* Mapping in a Human Knee Joint: A Feasibility Study,” NMR in Biomedicine 33, no. 11 (2020): e4391, 10.1002/nbm.4391. 32761692 PMC7584401

[31] V. Juras , S. Zbyn , C. Pressl , et al., “Regional Variations of T2 * in Healthy and Pathologic Achilles Tendon in Vivo at 7 Tesla: Preliminary Results,” Magnetic Resonance in Medicine 68, no. 5 (2012): 1607–1613, 10.1002/mrm.24136. 22851221

[32] J. Liu , A. Nazaran , Y. Ma , et al., “Single‐ and Bicomponent Analyses of T2* Relaxation in Knee Tendon and Ligament by Using 3D Ultrashort Echo Time Cones (UTE Cones) Magnetic Resonance Imaging,” BioMed Research International 2019, no. 1 (2019): 8597423, 10.1155/2019/8597423. 30906782 PMC6398070

[33] R. Kijowski , J. J. Wilson , and F. Liu , “Bicomponent Ultrashort Echo Time Analysis for Assessment of Patients With Patellar Tendinopathy,” Magnetic Resonance Imaging 46, no. 5 (2017): 1441–1447, 10.1002/jmri.25689. PMC5587350 28263448

[34] V. Juras , S. Apprich , P. Szomolanyi , O. Bieri , X. Deligianni , and S. Trattnig , “Bi‐Exponential T2* Analysis of Healthy and Diseased Achilles Tendons: An in Vivo Preliminary Magnetic Resonance Study and Correlation With Clinical Score,” European Radiology 23, no. 10 (2013): 2814–2822, 10.1007/s00330-013-2897-8. 23760303 PMC3769589

[35] X. Lu , S. Jerban , L. Wan , et al., “Three‐Dimensional Ultrashort Echo Time Imaging With Tricomponent Analysis for Human Cortical Bone,” Magnetic Resonance in Medicine 82, no. 1 (2019): 348–355, 10.1002/mrm.27718. 30847989 PMC6491227

[36] S. Jerban , X. Lu , E. W. Dorthe , et al., “Correlations of Cortical Bone Microstructural and Mechanical Properties With Water Proton Fractions Obtained From Ultrashort Echo Time (UTE) MRI Tricomponent T2* Model,” NMR in Biomedicine 33, no. 3 (2020): e4233, 10.1002/nbm.4233. 31820518 PMC7161421

[37] P. Meunier , J. Aaron , C. Edouard , and G. VlGNON , “Osteoporosis and the Replacement of Cell Populations of the Marrow by Adipose Tissue: A Quantitative Study of 84 Iliac Bone Biopsies,” Clinical Orthopaedics and Related Research 80 (1971): 147–154. 5133320 10.1097/00003086-197110000-00021

[38] T. Y. Kim and A. L. Schafer , “Diabetes and Bone Marrow Adiposity,” Current Osteoporosis Reports 14, no. 6 (2016): 337–344, 10.1007/s11914-016-0336-x. 27714580 PMC5126977

[39] T. L. Nickolas , E. Stein , A. Cohen , et al., “Bone Mass and Microarchitecture in CKD Patients With Fracture,” Journal of the American Society of Nephrology 21, no. 8 (2010): 1371–1380, 10.1681/ASN.2009121208. 20395370 PMC2938588

[40] M. J. Devlin and C. J. Rosen , “The Bone–Fat Interface: Basic and Clinical Implications of Marrow Adiposity,” Lancet Diabetes & Endocrinology 3, no. 2 (2015): 141–147, 10.1016/S2213-8587(14)70007-5. 24731667 PMC4138282

