# Supplementary material for: Bicomponent Mapping of Cortical Bone Using a New Interleaved UTE Imaging Sequence
Source: Magn Reson Med. 2026 Jan 4;95(5):2755–64. doi: 10.1002/mrm.70245 (PMC12854791; doi:10.1002/mrm.70245)
Supplement: Supplementary file 1 — Table S1. Coefficient of variables (CoV) of the voxel‐wise measurements of T2* of the short T2 component (T2s*) and the fraction of the short T2 component (Fs) with and without fixing the T2* of the long T2 component (T2L*). The voxel‐wise measurements are shown in Figure 3 (fix) and Figure S1 (no fix). Figure S1. ROI‐based Bicomponent T2* fitting results from the tibial midshaft cortical bone of a 37‐year‐old male shown in Figure 3. Red arrows indicate signal oscillations from separate dual‐echo scans with and without registration. Figure S2. Comparison of standard deviations of voxel‐wise measurements of (A) T2s* fraction and (B) T2s* from interleaved and separate dual‐echo scans. Interleaved scan shows reduced variances in voxel‐wise mapping of bicomponent model parameters in general. The reduction of variances in the T2s* fraction measurement from the interleaved scan is statistically significant (p = 0.0342) compared to the results from separate scans. Figure S3. Bicomponent parameter maps from a 37‐year‐old male healthy subject's tibial midshaft with unfixed long T2* (T2L*) for fitting. In the bicomponent fitting, the lower and upper boundary of T2L* was set to 0 and 10 ms, respectively. Compared to the fitting results with fixed T2L* of 4 ms shown in Figure 3, the overall range of fitting results is similar but more unstable fitting. Nevertheless, the interleaved sequence still shows more stable fitting results compared to the separate dual‐echo acquisition with (Separate‐Reg) and without (Separate) image registration (T2s* = T2* of short T2 component, Fs = Fraction of short T2 component). Figure S4. Voxel‐wise bicomponent fitting results from a bovine cortical bone scan. The separate dual‐echo and interleaved scans yield highly consistent and relatively homogeneous T2s* and Fs (T2s* fraction) measurements. The relative difference maps, along with the mean relative differences between the two scans, are also shown. Notably, the T2s* (5.3%) and Fs (1.6%) diffe [file MRM-95-2755-s001.docx]

**Supporting Information**

|  |  | Fix | No Fix |
| --- | --- | --- | --- |
| T_2s_* | Sep | 40.8 % | 63.6 % |
|  | Sep Reg | 44.4 % | 54.2 % |
|  | Inter | 33.3 % | 36.8 % |
| F_s_ | Sep | 21.4 % | 43.4 % |
|  | Sep Reg | 23.6 % | 39.1 % |
|  | Inter | 11.4 % | 23.2 % |

**Supporting Table S1**. Coefficient of variables (CoV) of the voxel-wise measurements of T_2_* of the short T_2_ component (T_2s_*) and the fraction of the short T_2_ component (F_s_) with and without fixing the T_2_* of the long T_2_ component (T_2L_*). The voxel-wise measurements are shown in Figure 3 (Fix) and Supporting Figure S1 (No Fix).

**
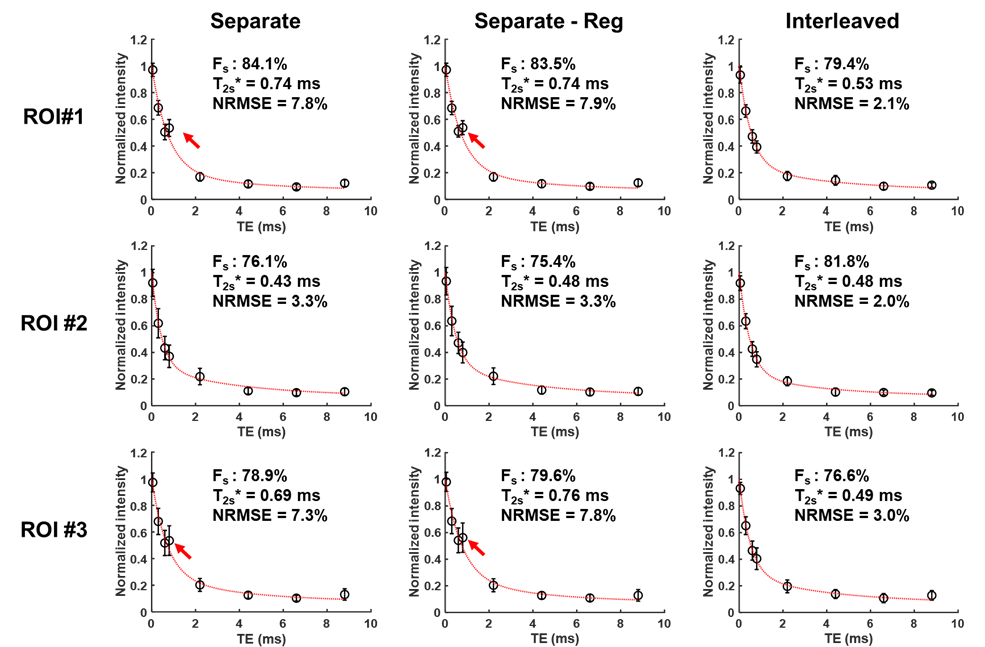
**

**Supporting Figure S1.** ROI-based Bicomponent T_2_* fitting results from the tibial midshaft cortical bone of a 37-year-old male shown in Figure 3. Red arrows indicate signal oscillations from separate dual-echo scans with and without registration.

**
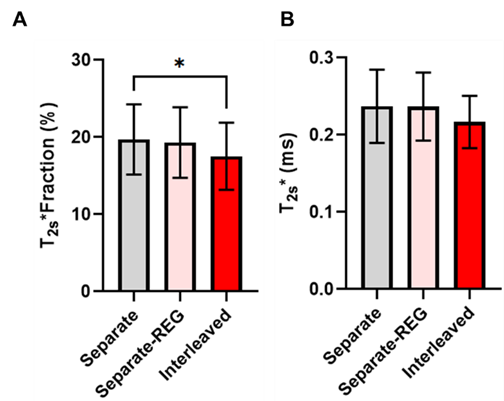
**

**Supporting Figure S2.** Comparison of standard deviations of voxel-wise measurements of (A) T_2s_* fraction and (B) T_2s_* from interleaved and separate dual-echo scans. Interleaved scan shows reduced variances in voxel-wise mapping of bicomponent model parameters in general. The reduction of variances in the T_2s_* fraction measurement from the interleaved scan is statistically significant (P = 0.0342) compared to the results from separate scans.

**
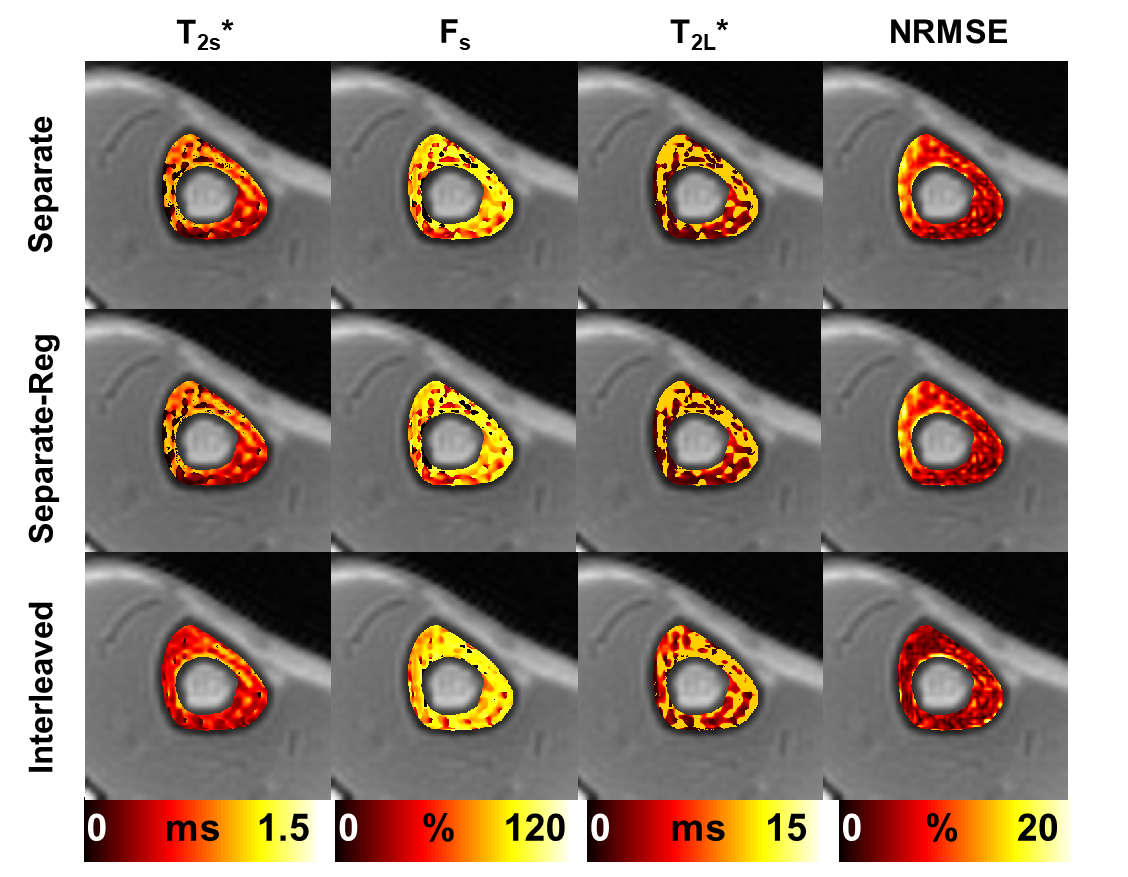
**

**Supporting Figure S3**. Bicomponent parameter maps from a 37-year-old male healthy subject’s tibial midshaft with unfixed long T_2_* (T_2L_*) for fitting. In the bicomponent fitting, the lower and upper boundary of T_2L_* was set to 0 and 10 ms, respectively. Compared to the fitting results with fixed T_2L_* of 4 ms shown in Figure 3, the overall range of fitting results is similar but more unstable fitting. Nevertheless, the interleaved sequence still shows more stable fitting results compared to the separate dual-echo acquisition with (Separate-Reg) and without (Separate) image registration. (T_2s_* = T_2_* of short T_2_ component, F_s_ = Fraction of short T_2_ component).


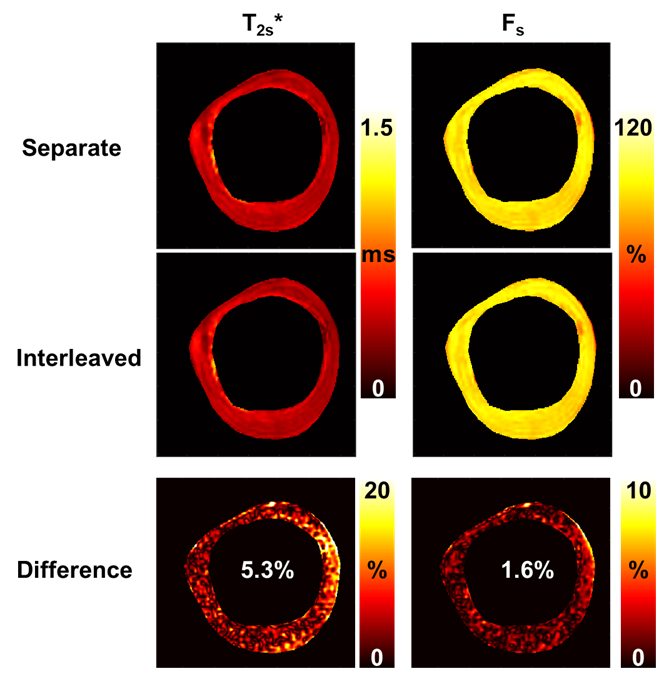


**Supporting Figure S4**. Voxel-wise bicomponent fitting results from a bovine cortical bone scan. The separate dual-echo and interleaved scans yield highly consistent and relatively homogeneous T_2s_* and F_s_ (T_2s_* fraction) measurements. The relative difference maps, along with the mean relative differences between the two scans, are also shown. Notably, the T_2s_* (5.3%) and F_s_ (1.6%) differences are substantially lower than the relative differences observed in vivo (T_2s_*: ~28%; F_s_: ~7%).


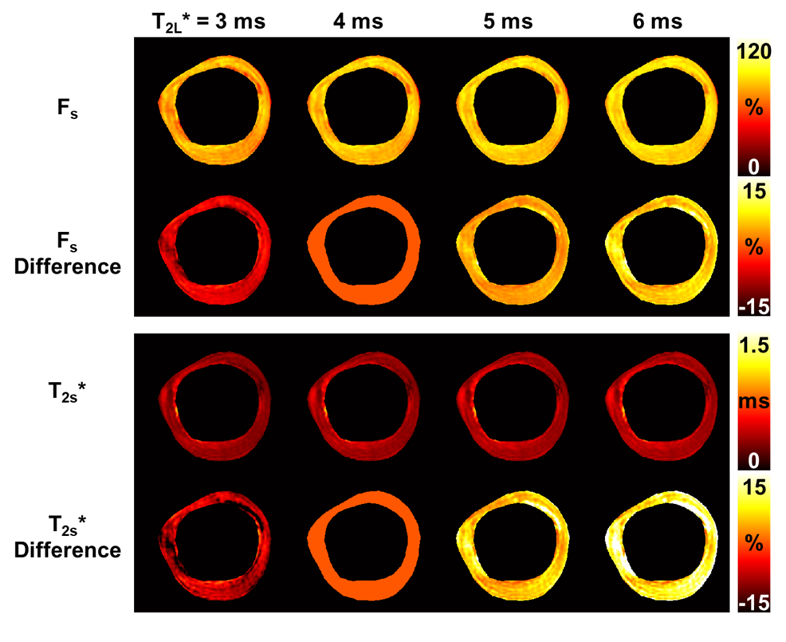


**Supporting Figure S5**. Voxel-wise bicomponent fitting of the interleaved ex vivo bovine bone scan with varying values of fixed T_2L_*. The differences were computed by subtracting the fitting results obtained with T_2L_* = 4 ms from those using other tested values, and then normalizing by the T_2L_* = 4 ms result. Overall, F_s_ and T_2s_* vary by less than ±10% when T_2L_* is varied from 3 to 6 ms, which is consistent with typical observations reported in previous bicomponent analysis studies.


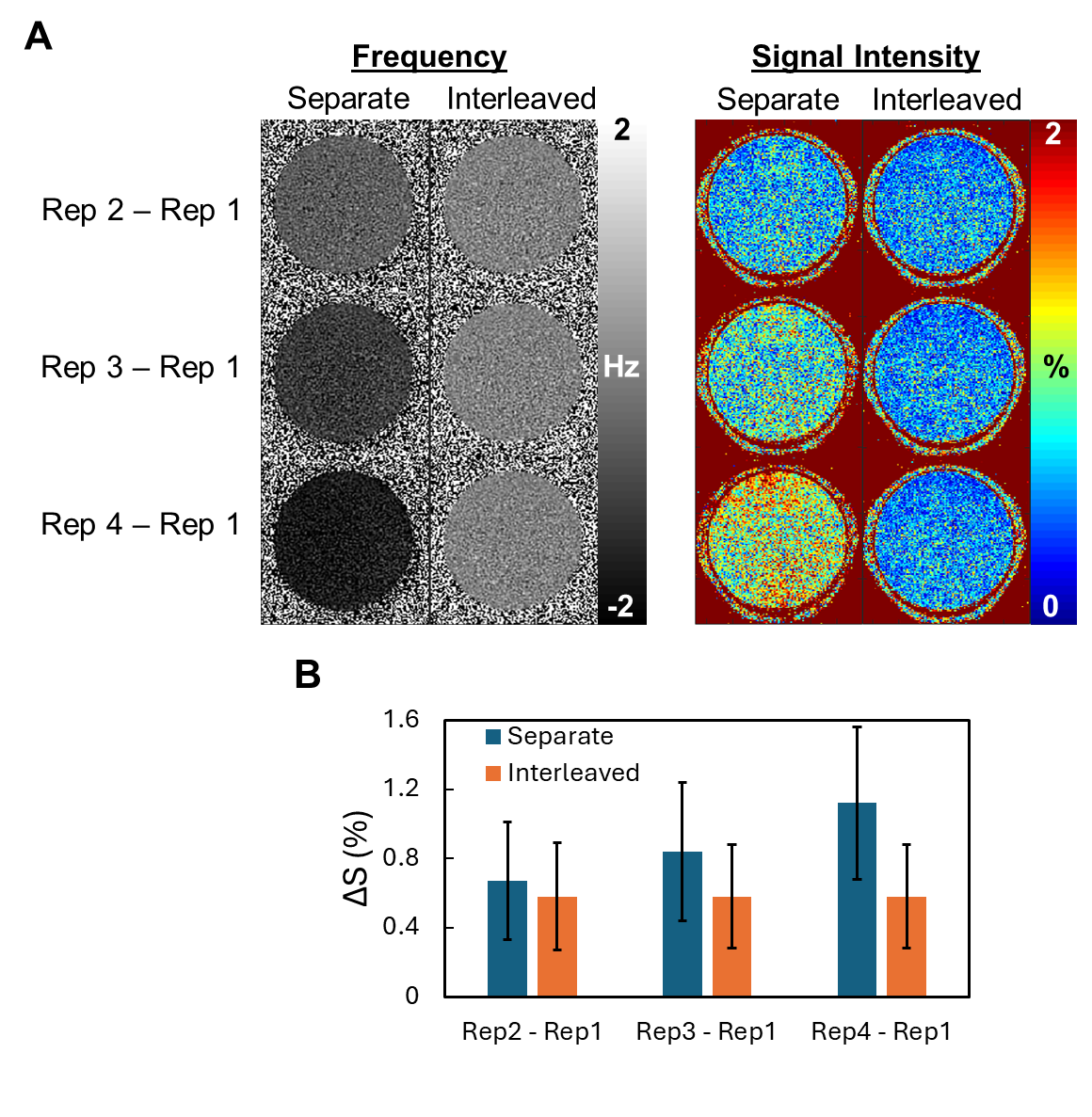


**Supporting Figure S6**. Evaluation of frequency and signal drift in interleaved and separate dual-echo scans. (A) The maps of frequency (left) and signal difference (right) between the first acquisition (Rep 1) and subsequent repeated scans (Rep 2, 3, 4) of a homogenous phantom. Note that the interleaved acquisition shows a consistent frequency and signal intensity between the first image and the subsequent repeat scans, whereas increasing frequency and signal differences over repeats are evident in separate dual-echo scans. (B) The signal drift measurements from an ROI encompassing the entire phantom.


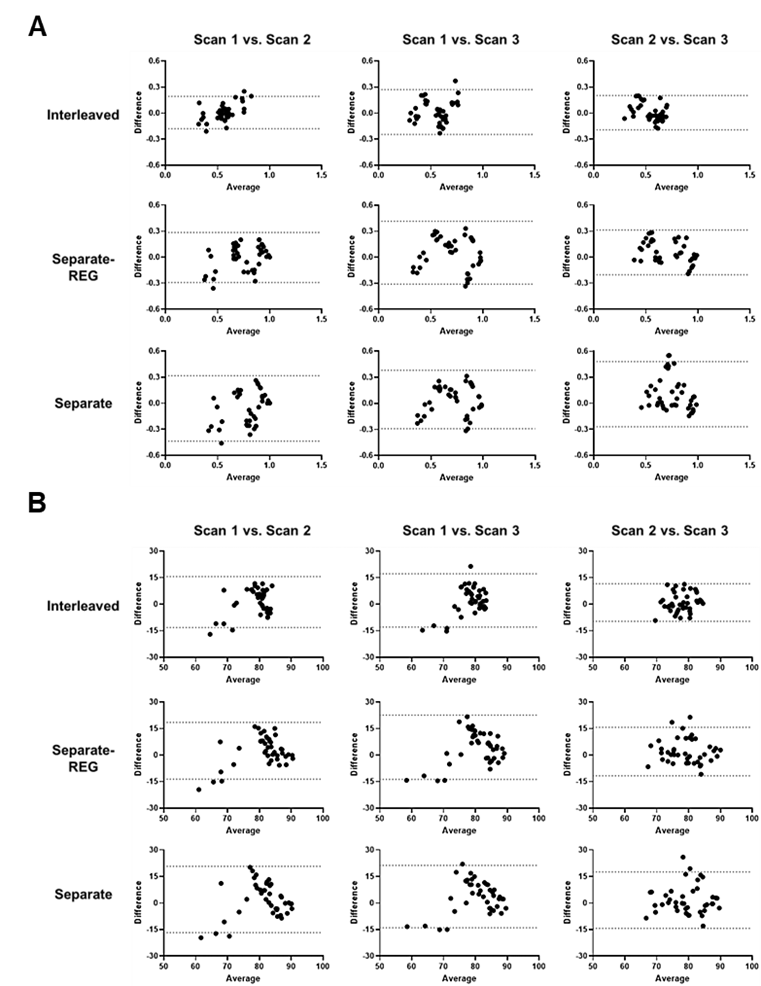


**Supporting Figure S7**. Bland-Altman plots of (A) T_2s_* and (B) T_2s_* fraction measurements from repeat scans of three subjects. Dashed lines indicate ±1.96 standard deviation. The fitted results from interleaved and separate dual-echo scans with and without registration show the measurements among repeat scans align well. The interleaved scans show smaller variations in the differences between the repeat scans compared to the separate dual-echo scans.
